# The Effects of Implementing the International Association of Diabetes and Pregnancy Study Groups Criteria for Diagnosing Gestational Diabetes on Maternal and Neonatal Outcomes

**DOI:** 10.1371/journal.pone.0122261

**Published:** 2015-03-10

**Authors:** Tai-Ho Hung, T’sang-T’ang Hsieh

**Affiliations:** 1 Department of Obstetrics and Gynecology, Chang Gung Memorial Hospital at Taipei, Taipei, Taiwan; 2 Department of Chinese Medicine, College of Medicine, Chang Gung University, Taoyuan, Taiwan; Virgen Macarena University Hospital, School of Medicine, University of Seville, SPAIN

## Abstract

**Background:**

In 2010, the International Association of the Diabetes and Pregnancy Study Groups (IADPSG) recommended a new strategy for the screening and diagnosis of gestational diabetes mellitus (GDM). However, no study has indicated that adopting the IADPSG recommendations improves perinatal outcomes. The objective of this study was to evaluate the effects of implementing the IADPSG criteria for diagnosing GDM on maternal and neonatal outcomes.

**Methodology/Principal Findings:**

Previously, we used a two-step approach (a 1-h, 50-g glucose challenge test followed by a 3-h, 100-g glucose tolerance test when indicated) to screen for and diagnose GDM. In July 2011, we adopted the IADPSG recommendations in our routine obstetric care. In this study, we retrospectively compared the rates of various maternal and neonatal outcomes in all women who delivered after 24 weeks of gestation during the periods before (P1, between January 1, 2009 and December 31, 2010) and after (P2, between January 1, 2012 and December 31, 2013) the IADPSG criteria were implemented. Pregnancies complicated by multiple gestations, fetal chromosomal or structural anomalies, and pre-pregnancy diabetes mellitus were excluded. Our results showed that the incidence of GDM increased from 4.6% using the two-step method to 12.4% using the IADPSG criteria. Compared to the women in P1, the women in P2 experienced less weight gain during pregnancy, lower birth weights, shorter labor courses, and lower rates of macrosomia (<4000 g) and large-for-gestational age (LGA) infants. P2 was a significant independent factor against macrosomia (adjusted odds ratio [OR] 0.63, 95% confidence interval [CI] 0.43–0.90) and LGA (adjusted OR 0.74, 95% CI 0.61–0.89) after multivariable logistic regression analysis.

**Conclusions/Significance:**

The adoption of the IADPSG criteria for diagnosis of GDM was associated with significant reductions in maternal weight gain during pregnancy, birth weights, and the rates of macrosomia and LGA.

## Introduction

Women with gestational diabetes mellitus (GDM) are more likely to require operative vaginal and cesarean deliveries and are at risk for obstetrical complications, including preeclampsia, large-for-gestational age (LGA) infants, macrosomia, shoulder dystocia, and birth injury [1]. Neonates of GDM pregnancies are also at risk for hypoglycemia, hyperbilirubinemia, and respiratory distress syndrome. Nevertheless, screening and diagnostic tests for GDM are not uniform worldwide; various strategies have been endorsed by different professional organizations, such as the American College of Obstetricians & Gynecologists (ACOG) [2] and the World Health Organization (WHO) [3]. Therefore, it is difficult to compare GDM trends across populations and over time and to apply the results obtained from research in one setting to patients in another setting. Moreover, the thresholds for most of these GDM diagnostic methods are based on two standard deviations above the mean blood glucose levels or simply based on criteria used in non-pregnant women, which are not directly related to perinatal outcomes.

The Hyperglycemia and Adverse Pregnancy Outcome (HAPO) Study was a large multicenter multinational study of values obtained from blinded, 75 g, 2 h oral glucose tolerance tests (OGTTs) [4]. The study demonstrated a continuum of risk for maternal glucose levels and adverse pregnancy outcomes. Following the release of the HAPO Study, the International Association of the Diabetes and Pregnancy Study Groups (IADPSG) made recommendations in an attempt to reach international uniformity and consensus on the diagnostic criteria for GDM with respect to its value in predicting adverse pregnancy outcomes [5]. The IADPSG recommends diagnosing GDM when one or more of the following plasma glucose levels (based on the 75-g, 2-h, OGTT) was met or exceeded: fasting, 92 mg/dL; 1-h, 180 mg/dL; or 2-h, 153 mg/dL. These revised, lower cut-off values were derived from the HAPO study and represent an odds ratio for adverse pregnancy outcomes of 1.75 compared with women without GDM. Thus far, the IADPSG recommendations have been adopted by the American Diabetes Association [6] and are being considered worldwide [7,8].

However, several concerns have been raised about the IADPSG recommended criteria for diagnosing GDM [9–11]. According to the HAPO Study results [4], the predicted prevalence of an abnormal test would diagnose nearly 18% of all pregnant women with GDM, which would cause excessive healthcare costs and workloads. These drawbacks are acceptable and justified only if maternal and neonatal health are improved. Nevertheless, no randomized controlled trials have shown that adopting the IADPSG recommendations would improve perinatal outcomes.

At Chang Gung Memorial Hospital, we previously performed a two-step approach to screen for and diagnose GDM, as recommended by ACOG [2]. In July 2011, we adopted the IADPSG recommendations in our routine obstetric care. This change provided us with an opportunity to compare the incidence of GDM and the rates of various maternal and neonatal outcomes before and after implementing the IADPSG criteria for the diagnosis of GDM.

## Materials and Methods

A retrospective cohort study of women who gave birth at Chang Gung Memorial Hospital, Taipei, Taiwan, was performed between January 1, 2009 and December 31, 2013. Study data were obtained from a computerized obstetrics database, which included demographic characteristics, medical and obstetric histories, as well as information on the course of the index pregnancy and perinatal outcomes [12].

Data in this database were collected by trained personnel through daily extraction from medical and delivery records, with a postpartum interview, if necessary, to collect supplemental information. Audits of these data were routinely performed at weekly departmental meetings. The study was approved by the Institutional Review Board of Chang Gung Memorial Hospital (No. 103-2304B); the review board also determined that informed consent was not required due to the retrospective design of the study and the fact that all participants were anonymized.

Prior to July 2011, we used a two-step approach to screen and diagnose GDM: first, a non-fasting 50-g, 1-h glucose challenge test (GCT) was performed for all non-diabetic women [2]. If the results were ≥140 mg/dL, a 100-g, 3-h OGTT was then performed. The OGTT thresholds were as follows: fasting glucose, 95 mg/dL; 1-h, 180 mg/dL; 2-h, 155 mg/dL; and 3-h, 140 mg/dL. The GDM diagnosis was established by two or more abnormal values on the OGTT.

In January 2011, the IADPSG screening strategy was introduced and recommended by the Taiwan Society of Perinatology. Following thorough discussion, by consensus, the department decided to replace the two-step method with the IADPSG one-step method as our routine method of GDM screening and diagnosis. This implementation formally began on July 1, 2011.

At Chang Gung Memorial Hospital, universal screening for GDM is performed on all pregnant women. The screening is usually performed between 24 and 28 weeks of gestation or at the first prenatal visit in high-risk women. If GDM was diagnosed, either by the two-step or one-step method, the woman was given dietary and lifestyle modification advice and underwent regular monitoring of blood glucose levels. Insulin treatment was indicated if medical nutritional therapy failed to consistently maintain a fasting glucose level <95 mg/dL and a 2-h postprandial level <120 mg/dL.

For the purposes of our research, the study population was divided into two groups; women who delivered during the period before (period 1 [P1], between January 1, 2009 and December 31, 2010) and after (period 2 [P2], between January 1, 2012 and December 31, 2013) the program was implemented. Deliveries between January 1 and December 31, 2011 were excluded from the analysis to avoid the heterogeneity of the screening methods during the transition period.

We analyzed all deliveries after 24 weeks of gestation, as elective abortion is permitted in Taiwan before 24 weeks of pregnancy. Pregnancies complicated by multiple gestations, fetal chromosomal or structural anomalies, and pre-pregnancy diabetes mellitus were also excluded. To avoid concerns regarding the correlated nature of the pregnancy outcomes, we also limited the analysis to the first pregnancy of a given woman who was seen during the study period.

Our primary outcomes were macrosomia (defined as >4000 g birth weight) and LGA (defined as a birth weight above the 90th percentile for the mean weight corrected for fetal sex and gestational age) [13]. The rates of additional maternal and neonatal outcomes were also compared between the two groups. These rates included GDM, preeclampsia, placenta previa, placental abruption, placenta accreta, oligohydramnios (an amniotic fluid index [AFI] <5 cm), polyhydramnios (AFI >24 cm), premature rupture of membranes, postpartum hemorrhage, fetal death after 24 weeks of gestation, 1-minute and 5-minute Apgar scores below 7, admission to the neonatal intensive care unit (NICU), neonatal death within 28 days of birth, preterm birth before 37 weeks of gestation, low birth weight (<2500 g), and small-for-gestational age (SGA, defined as a birth weight below the 10th percentile for the mean weight corrected for fetal sex and gestational age).

Statistical analyses were performed using SPSS software, version 20.0 (SPSS Inc., Armonk, NY, USA). Continuous variable data were tested for normality using the Kolmogorov-Smirnov test and are presented as medians (interquartile range) because they were not normally distributed. Categorical variables were calculated as a number and rate (%). Continuous parameters were compared between the groups using the Mann-Whitney *U* test and categorical variables were compared using the γ^2^ test. A *P* value of <0.05 was considered to be statistically significant.

To further clarify the association between adverse pregnancy outcomes and different time periods, a multiple logistic regression analysis was performed. Those maternal characteristics that differed significantly in prevalence between the two cohorts and additional factors that we considered important to the growth of the fetus were included in the multiple logistic regression to control for confounding effects. These factors included maternal age, parity, pre-pregnancy body mass index (BMI), conception methods, cigarette smoking, chronic hypertension, fetal sex, placenta previa, and GDM. A backward elimination process was used to obtain the final regression model, with *P*<0.05 as the selection criterion for stepwise procedures. Adjusted odds ratios (OR) and 95% confidence intervals (CI) were calculated to describe the relative risk.

## Results

After excluding the deliveries that occurred before 24 weeks of gestation (n = 193) and the pregnancies that were complicated by multiple gestations (n = 298), fetal anomalies (n = 47), and pre-pregnancy diabetes mellitus (n = 19), there were 3056 and 3641 deliveries in P1 and P2, respectively. The prevalence of GDM increased from 4.6% in P1 to 12.4% in P2 (*P*<0.001), and the rate of insulin therapy was 7.3% in P1 and 3.9% in P2.

Table 1 shows the maternal and pregnancy characteristics of the study groups before (P1) and after (P2) the IADPSG criteria were implemented. Compared to the women in P1, higher rates of women older than 34 years (34.3% vs. 30.8%, *P* = 0.002) and primiparity (58.3% vs. 53.5%, *P*<0.001) were noted in P2. Notably, the women in P2 had less median weight gain during pregnancy compared to the women in P1 (13.2 vs. 13.6 kg, *P* = 0.001).

**Table 1:** Maternal and pregnancy characteristics in the study groups before (P1) and after (P2) the implementation of the IADPSG criteria.

Characteristics	P1 (n = 3056)	P2 (n = 3641)	*P*
Age (y)			
<20	7 (0.2%)	9 (0.2%)	0.880
20–34	2109 (69.0%)	2382 (65.4%)	0.002
>34	940 (30.8%)	1250 (34.3%)	0.002
Pre-pregnancy BMI (kg/m^2^)			
<19.8	1042 (34.1%)	1318 (36.2%)	0.076
19.8–24.2	1584 (51.8%)	1808 (49.7%)	0.077
>24.2	430 (14.1%)	515 (14.1%)	0.944
Weight gain during pregnancy (kg)	13.6 (11.0–16.1)	13.2 (10.7–15.8)	0.001
Primiparity	1636 (53.5%)	2123 (58.3%)	<0.001
Prior fetal death	2 (0.1%)	3 (0.1%)	0.880
Prior preterm birth	1 (0.0%)	1 (0.0%)	0.901
Conception assisted by reproductive technology	35 (1.1%)	58 (1.6%)	0.142
Cigarette smoking	7 (0.2%)	6 (0.2%)	0.588
Amniocentesis	1116 (36.5%)	1396 (38.3%)	0.128
Chronic hypertension	7 (0.2%)	9 (0.2%)	0.890
Hypothyroidism	2 (0.1%)	7 (0.2%)	0.194
Hyperthyroidism	6 (0.2%)	15 (0.4%)	0.129
Gestational diabetes mellitus	141 (4.6%)	453 (12.4%)	< 0.001
Group B streptococcal colonization	440 (14.4%)	502 (13.8%)	0.481
Cord entanglement	643 (21.0%)	697 (19.1%)	0.053
Male fetus	1569 (51.3%)	1895 (52.0%)	0.572

Data are presented as a number (%) or median (interquartile range).

BMI, body mass index.

*P* values are based on the γ^2^ test or Mann-Whitney *U* test.

Table 2 shows the delivery characteristics of the study populations. There were no differences between the rates of operative vaginal delivery and primary cesarean delivery. Nevertheless, the women in P2 had a shorter duration of the first stage of labor (130 vs. 150 min, *P*<0.001), and they were less likely to have labor augmentation compared to the women in P1 (32.9% vs. 43.2%, *P*<0.001).

**Table 2:** Delivery characteristics in the study groups before (P1) and after (P2) the implementation of the IADPSG criteria.

Characteristics	P1 (n = 3056)	P2 (n = 3641)	*P*
Induction of labor	527 (17.2%)	573 (15.7%)	0.098
Augmentation of labor	1321 (43.2%)	1199 (32.9%)	<0.001
Spontaneous vaginal delivery	1948 (63.7%)	2252 (61.9%)	0.116
Operative vaginal delivery	51 (1.7%)	65 (1.8%)	0.778
First stage of labor (min)^a^	150 (70–270)	130 (60–250)	<0.001
Second stage of labor (min)^a^	35 (18–66)	34 (18–71)	0.081
Cesarean delivery	1057 (34.6%)	1324 (36.4%)	0.131
Repeat cesarean section	390 (12.8%)	483 (13.3%)	0.560
Primary cesarean section^b^	667 (21.8%)	841 (23.1%)	0.218
Dysfunctional labor	348 (11.4%)	443 (12.2%)	0.342
Fetal malpresentation	200 (6.5%)	261 (7.2%)	0.333
Non-reassuring FHR	118 (3.9%)	160 (4.4%)	0.296
Prior hysterotomy	38 (1.2%)	41 (1.1%)	0.733
Placenta previa	52 (1.7%)	66 (1.8%)	0.780
Macrosomia	20 (0.7%)	16 (0.4%)	0.244
Elective	145 (4.7%)	160 (4.4%)	0.518

Data are presented as a number (%) or median (interquartile range).

FHR, fetal heart rate.

*P* values are based on the γ^2^ test or Mann-Whitney *U* test.

^a^, Labor durations for women with spontaneous or operative vaginal deliveries.

^b^, Indications for primary cesarean section. Some women had more than one indication for cesarean delivery.

The pregnancy outcomes of women who delivered during these two periods are summarized in Table 3. Women in both P1 and P2 had similar gestational weeks at delivery and neonatal lengths. However, the women in P2 had lower median birth weights (3120 vs. 3158 g, *P*<0.001) but greater median neonatal head circumferences (34.0 vs. 33.5 cm, *P*<0.001) compared to the women in P1. A lower median neonatal weight-to-length ratio was also noted in the women in P2 (6.2 vs. 6.3, *P* = 0.01). Moreover, the rates of macrosomia (1.5% vs. 2.3%, *P* = 0.037) and LGA (6.3% vs. 7.8%, *P* = 0.016) were significantly lower in the women in P2 compared with those rates in the women in P1. There were no differences in the incidences of other adverse neonatal outcomes, such as preterm birth, low birth weight, SGA, low Apgar scores, fetal or neonatal death, and NICU admission between these two groups. Likewise, no differences in the rates of a variety of pregnancy complications, such as preeclampsia, placenta accreta, placental abruption, oligohydramnios, polyhydramnios, premature rupture of membranes, and postpartum hemorrhage, were noted between the two groups.

**Table 3:** Pregnancy outcomes in the study groups before (P1) and after (P2) the implementation of the IADPSG criteria.

Variable	P1 (n = 3056)	P2 (n = 3641)	*P*
Gestational age (wk)	39 (38–40)	39 (38–40)	0.063
Birth weight (g)	3158 (2895–3430)	3120 (2855–3380)	<0.001
Fetal head circumference (cm)	33.5 (32.5–34.5)	34.0 (32.5–35.0)	<0.001
Fetal length (cm)	50.0 (49.0–51.5)	50.0 (49.0–52.0)	0.686
Fetal weight-to-length ratio (kg/m)	6.3 (5.9–6.7)	6.2 (5.8–6.6)	<0.001
Preterm birth (<37 wk)	253 (8.3%)	314 (8.6%)	0.660
Birth weight <2500 g	203 (6.6%)	272 (7.5%)	0.197
Birth weight >4000 g	69 (2.3%)	56 (1.5%)	0.037
Small-for-gestational age	303 (9.9%)	404 (11.1%)	0.120
Large-for-gestational age	238 (7.8%)	228 (6.3%)	0.016
1-minute Apgar score <7	46 (1.5%)	41 (1.1%)	0.194
5-minute Apgar score <7	8 (0.3%)	10 (0.3%)	0.919
NICU admission	79 (2.6%)	93 (2.6%)	0.938
Neonatal death	1 (0.0%)	4 (0.1%)	0.384
Fetal death	6 (0.2%)	3 (0.1%)	0.316
Preeclampsia	54 (1.8%)	85 (2.3%)	0.121
Placenta accreta	13 (0.4%)	17 (0.5%)	0.856
Placental abruption	49 (1.6%)	70 (1.9%)	0.354
Oligohydramnios	39 (1.3%)	39 (1.1%)	0.493
Polyhydramnios	5 (0.2%)	10 (0.3%)	0.440
Premature rupture of membranes	50 (1.6%)	58 (1.6%)	0.923
Postpartum hemorrhage	49 (1.6%)	57 (1.6%)	0.922

Data are presented as a number (%) or median (interquartile range).

NICU, neonatal intensive care unit.

*P* values are based on the γ^2^ test or Mann-Whitney *U* test.

To further clarify the associations between macrosomia, LGA, and the different time periods, a multiple logistic regression analysis was performed, controlling for confounding effects of maternal age, parity, pre-pregnancy BMI, conception methods, amniocentesis, cigarette smoking, chronic hypertension, fetal gender, placenta previa, and GDM. In P2, the women were less likely to have newborns with macrosomia (adjusted OR 0.63, 95% CI 0.43–0.90) and LGA (adjusted OR 0.74, 95% CI 0.61–0.89) compared to the women in P1. The other outcomes were not statistically different between the two groups of women. The unadjusted and adjusted ORs and associated 95% CIs are presented in S1 Table (see supporting information).

## Discussion

The arguments in favor of adopting the IADPSG criteria to diagnose GDM include the thresholds developed based on pregnancy outcomes, greater convenience for the care providers and patients by eliminating the screening phase, and earlier diagnosis and treatment of glucose intolerance during pregnancy, in hopes of more consistent care and improved organization of GDM research across different populations [9–11]. Nevertheless, little evidence indicates that such a change would lead to better pregnancy outcomes.

A few studies have evaluated the effects of the IADPSG criteria on maternal and neonatal outcomes. These studies retrospectively analyzed pregnancy outcomes in the same cohort of women who were previously classified as normal by other GDM criteria and, subsequently, diagnosed with GDM, according to the IADPSG criteria. O’Sullivan et al. found that women who were diagnosed with normal glucose tolerance using the WHO criteria but diagnosed with GDM using IADPSG criteria had a significantly greater risk of gestational hypertension, preeclampsia, cesarean delivery, macrosomia, LGA, and NICU admission of the newborns compared to the women diagnosed with normal glucose tolerance using the IADPSG criteria [14]. Lapolla et al. also found that increased rates of cesarean delivery and fetal morbidities occurred in the women reclassified with GDM using the IADPSG criteria compared with the women classified as normal, although no between-group differences were observed in birth weight and rates of macrosomia and LGA [15]. Likewise, Benhalima et al. observed that women who were previously classified as normal using the Carpenter and Coustan criteria and then, subsequently, classified as GDM using the IADPSG criteria had increased rates of cesarean delivery and shoulder dystocia compared to the women with normal glucose tolerance, despite similar rates of macrosomia and LGA [16]. In contrast, Bodmer-Roy et al. reported that the women who were classified as non-diabetic using the Canada Diabetes Association criteria but who were considered to have GDM using the IADPSG criteria had similar pregnancy outcomes as the women without GDM [17]. More recently, in a randomized trial, Sevket et al. observed that the prevalence of GDM in the women diagnosed using the IADPSG criteria was 14.5% compared to 6% in the women diagnosed with GDM defined using the two-step method [18]. Additionally, the women with positive 50-g GCT results but negative 3-h, 100-g OGTT results had a greater risk of preeclampsia and polyhydramnios than the women who were defined as having normal glucose tolerance using the IADPSG criteria. Together, although not consistent throughout all studies, most reports indicated that women previously classified as normal using other GDM criteria and then, subsequently, diagnosed with GDM using the IADPSG criteria were at an increased risk for various adverse perinatal outcomes compared to the women with normal glucose tolerance, as defined by the IADPSG criteria. Of note, none of these studies investigated the differential treatment effect between women with GDM diagnosed using the IADPSG criteria and women with GDM diagnosed using other criteria.

From a different perspective, we evaluated the effects of adopting the IADPSG criteria on maternal and neonatal outcomes using a sequential design. Our analysis showed that in the period following the implementation of the IADPSG criteria, women had less weight gain during pregnancy and lower birth weights compared to women in the period before the program was implemented. These women also had newborns with significantly lower rates of macrosomia and LGA and a shorter labor course, despite showing no difference in the rates of preeclampsia and operative vaginal or cesarean delivery. These results suggest that implementing the IADPSG criteria is associated with reduced maternal weight gain during pregnancy as well as decreased birth weight and a lower incidences of macrosomia and LGA. The mechanisms behind these findings are unclear. It is possible that some women with glucose intolerance who would have been regarded as non-diabetic if the two-step method was used were identified by the IADPSG criteria and, therefore, were treated as GDM, thus producing better pregnancy outcomes. Indeed, the treatment of women with GDM is associated with reductions in the fetal size (i.e., birth weight and fat mass) and in the rates of adverse perinatal outcomes, including macrosomia, LGA, shoulder dystocia, cesarean delivery, preeclampsia, and gestational hypertension [19–21].

Alternatively, it might be argued that improved care over the time periods of the study might have contributed to the improved outcomes. This scenario is unlikely, as the clinical structure of the department did not change significantly, and there were no additional changes in obstetric care during the time period that would be expected to confound these results.

Compared to P1, there were more deliveries, more women aged more than 34 years and more primiparity in P2. These changes are likely related to Chinese zodiacal preferences, as 2010 was the year of the Tiger and 2012 was the year of the Dragon. Women favor having babies during the Dragon Year rather than the Tiger Year as a result of Chinese cultural preferences and folklore beliefs.

In this study, the adoption of the IADPSG criteria was also noted to have an effect on fetal anthropometry. In addition to birth weight, the neonates in the period after implementation of the IADPSG criteria had a significantly lower weight-to-length ratio but a larger head circumference compared to the neonates in the period before the program was implemented. Weight-to-length ratio is a readily available birth index that correlates well with the total skinfold thickness, thus, reflecting the intrauterine nutritional state [22]. Again, this finding aligns with a prior study that showed that treatment of mild GDM, defined as a fasting glucose level <95 mg/dL and two or three timed glucose measurements that exceeded established thresholds (1-h, 180 mg/dL; 2-h, 155 mg/dL; and 3-h, 140 mg/dL) was associated with a significant reduction in birth weight and neonatal fat mass compared with usual care [20].

Although they reached statistical significance, the magnitude of changes in maternal weight gain during pregnancy, birth weight, and the fetal weight-to-length ratio between women in P1 and P2 was minor in this study in comparison with the increase in the rate of GDM. The clinical significance of these changes remains elusive. Moreover, it is not clear whether similar changes would be found in other populations or if the magnitude of changes would be different in populations with more obese women or with different nutritional behavior. Further studies are needed to verify our findings and to investigate the clinical significance of these changes.

The current study had some limitations. First, the study is limited by its observational and retrospective design. Therefore, information on neonatal hypoglycemia or hyperbilirubinemia is lacking. Second, there might be one or more unmeasured confounders that may have contributed to the decline in the rates of adverse pregnancy outcomes following the adoption of the IADPSG criteria. Nevertheless, we were able to control for the key measured confounders for macrosomia and LGA using multiple logistic regression analysis. Third, this study has a limited sample size of some important but rare pregnancy complications, such as preeclampsia [23] and shoulder dystocia [24], in Asian populations.

As expected, the adoption of the IADPSG criteria led to a nearly three-fold increase in the incidence of GDM (4.6% in P1 vs. 12.4% in P2) in our population; the average number of women with GDM increased from 6 per month in P1 to 19 per month in P2, as our hospital has a moderate obstetrical volume (150 deliveries per month). The addition of 13 women was still well managed by our diabetes educators and dieticians. Nevertheless, such an increase in the rate of GDM will generate a greater workload for obstetricians, endocrinologists, and dietician services in hospitals with high obstetrical volume and may raise concerns regarding the cost-effectiveness of the IADPSG recommendations for the entire health care system. Another study limitation is that there was no comparison of the cost-effectiveness between the two-step method and the IADPSG criteria. However, two recent studies have shown some economic benefits. Werner et al. found that the IADPSG recommendations for GDM screening and diagnosis are more cost-effective than the two-step method, however, only if post-delivery consultation and intervention are provided to reduce the frequency of type 2 diabetes mellitus [25]. Similarly, Mission et al. found that the IADPSG recommendations are expansive but cost-effective in improving maternal and neonatal outcomes [26]. Nonetheless, how the health care system will provide expanded care to this patient group is an important issue that must be examined.

## Conclusions

In summary, this sequential study showed that adopting the IADPSG criteria for GDM diagnosis led to a significant reduction in maternal weight gain during pregnancy, birth weight, and rates of macrosomia and LGA. Because of the aforementioned limitations, further randomized controlled trials that simultaneously and directly compare perinatal outcomes using the current screening guidelines to the outcomes using the IADPSG guidelines are necessary.

## Supporting Information

S1 Table The association between adverse pregnancy outcomes and different time periods. (DOC)
