# Supplementary material for: The Effects of Implementing the International Association of Diabetes and Pregnancy Study Groups Criteria for Diagnosing Gestational Diabetes on Maternal and Neonatal Outcomes
Source: PLoS One. 2015 Mar 10;10(3):e0122261. doi: 10.1371/journal.pone.0122261 (PMC4355616; doi:10.1371/journal.pone.0122261)
Supplement: S1 Table — (DOC) [file pone.0122261.s001.doc]

**Table S1. The association between adverse pregnancy outcomes and different time periods**

| Outcome variable | P1 (n=3056) | P2 (n=3641) | Odds ratio (95% confidence interval) | | | |
| --- | --- | --- | --- | --- | --- | --- |
|  |  |  | Unadjusted | *P* | Adjusted^a^ | *P* |
| Preterm birth (<37 wk) | 253 (8.3%) | 314 (8.6%) | 1.04 (0.88-1.24) | 0.648 | 0.95 (0.80-1.14) | 0.604 |
| Birth weight <2500 g | 203 (6.6%) | 272 (7.5%) | 1.14 (0.94-1.37) | 0.189 | 1.05 (0.87-1.28) | 0.610 |
| Birth weight >4000 g | 69 (2.3%) | 56 (1.5%) | 0.68 (0.47-0.97) | 0.031 | 0.63 (0.43-0.90) | 0.012 |
| Small-for-gestational age | 303 (9.9%) | 404 (11.1%) | 1.13 (0.97-1.33) | 0.117 | 1.10 (0.93-1.29) | 0.271 |
| Large-for-gestational age | 238 (7.8%) | 228 (6.3%) | 0.79 (0.66-0.96) | 0.015 | 0.74 (0.61-0.89) | 0.002 |
| 1-minute Apgar score <7 | 46 (1.5%) | 41 (1.1%) | 0.75 (0.49-1.14) | 0.174 | 0.72 (0.46-1.10) | 0.129 |
| 5-minute Apgar score <7 | 8 (0.3%) | 10 (0.3%) | 1.05 (0.41-2.66) | 0.919 | 1.05 (0.41-2.71) | 0.913 |
| NICU admission | 79 (2.6%) | 93 (2.6%) | 0.99 (0.73-1.34) | 0.937 | 0.93 (0.68-1.27) | 0.649 |
| Neonatal death | 1 (0.0%) | 4 (0.1%) | 3.36 (0.38-30.08) | 0.278 | 3.54 (0.39-31.77) | 0.259 |
| Fetal death | 6 (0.2%) | 3 (0.1%) | 0.42 (0.11-1.68) | 0.219 | 0.43 (0.11-1.72) | 0.233 |
| Preeclampsia | 54 (1.8%) | 85 (2.3%) | 1.33 (0.94-1.88) | 0.106 | 1.18 (0.83-1.69) | 0.366 |
| Placenta accreta | 13 (0.4%) | 17 (0.5%) | 1.10 (0.53-2.26) | 0.800 | 0.92 (0.44-1.95) | 0.831 |
| Placental abruption | 49 (1.6%) | 70 (1.9%) | 1.20 (0.83-1.74) | 0.325 | 1.22 (0.84-1.77) | 0.291 |
| Oligohydramnios | 39 (1.3%) | 39 (1.1%) | 0.84 (0.54-1.31) | 0.437 | 0.82 (0.52-1.29) | 0.394 |
| Polyhydramnios | 5 (0.2%) | 10 (0.3%) | 1.68 (0.57-4.92) | 0.344 | 1.36 (0.45-4.10) | 0.589 |
| Premature rupture of membranes | 50 (1.6%) | 58 (1.6%) | 0.97 (0.67-1.43) | 0.889 | 0.93 (0.63-1.37) | 0.722 |
| Postpartum hemorrhage | 49 (1.6%) | 57 (1.6%) | 0.98 (0.66-1.43) | 0.901 | 0.96 (0.65-1.42) | 0.839 |

^a^ Adjusted for maternal age, parity, pre-pregnancy body mass index, conception methods, cigarette smoking, chronic hypertension, fetal sex, placenta previa, and gestational diabetes mellitus.
